# Overexpressed NEDD8 as a potential therapeutic target in esophageal squamous cell carcinoma

**DOI:** 10.20892/j.issn.2095-3941.2020.0484

**Published:** 2021-03-18

**Authors:** Jingrong Xian, Shiwen Wang, Yanyu Jiang, Lihui Li, Lili Cai, Ping Chen, Yue Liu, Xiaofei Zeng, Guoan Chen, Chen Ding, Robert M. Hoffman, Lijun Jia, Hu Zhao, Yanmei Zhang

**Affiliations:** 1 Department of Laboratory Medicine, Huadong Hospital Affiliated to Fudan University, Shanghai 200040, China; 2 Cancer Institute, Longhua Hospital, Shanghai University of Traditional Chinese Medicine, Shanghai 200032, China; 3 Research Center on Aging and Medicine, Fudan University, Shanghai 200040, China; 4 Shanghai Key Laboratory of Clinical Geriatric Medicine, Shanghai 200040, China; 5 Department of Basic Science of Oncology, College of Basic Medical Sciences, Zhengzhou University, Collaborative Innovation Center of Henan Province for Cancer Chemoprevention, Zhengzhou 450001, China; 6 School of Medicine, Southern University of Science and Technology, Shenzhen 518055, China; 7 State Key Laboratory of Genetic Engineering, Human Phenome Institute, Institutes of Biomedical Sciences, School of Life Sciences, Zhongshan Hospital, Fudan University, Shanghai 200032, China; 8 State Key Laboratory of Cell Differentiation and Regulation, Henan International Joint Laboratory of Pulmonary Fibrosis, Henan Center for Outstanding Overseas Scientists of Pulmonary Fibrosis, College of Life Science, Institute of Biomedical Science, Henan Normal University, Xinxiang 453007, China; 9 Department of Surgery, University of California, San Diego 92101, USA; 10 Anticancer Inc., San Diego 92101, USA

**Keywords:** sNEDD8, esophageal squamous cell carcinoma, cullin-RING E3 ubiquitin ligases, apoptosis, anticancer target

## Abstract

**Objective:**

The hyperactivated neddylation pathway plays an important role in tumorigenesis and is emerging as a promising anticancer target. We aimed to study whether NEDD8 (neural precursor cell expressed, developmentally down-regulated 8) might serve as a therapeutic target in esophageal squamous cell carcinoma (ESCC).

**Methods:**

The clinical relevance of NEDD8 expression was evaluated by using The Cancer Genome Atlas (TCGA) database and tissue arrays. NEDD8-knockdown ESCC cells generated with the CRISPR/Cas9 system were used to explore the anticancer effects and mechanisms. Quantitative proteomic analysis was used to examine the variations in NEDD8 knockdown-induced biological pathways. The cell cycle and apoptosis were assessed with fluorescence activated cell sorting. A subcutaneous-transplantation mouse tumor model was established to investigate the anticancer potential of NEDD8 silencing *in vivo*.

**Results:**

NEDD8 was upregulated at both the mRNA and protein expression levels in ESCC, and NEDD8 overexpression was associated with poorer overall patient survival (mRNA level: *P* = 0.028, protein level: *P* = 0.026, log-rank test). Downregulation of NEDD8 significantly suppressed tumor growth both *in vitro* and *in vivo*. Quantitative proteomic analysis revealed that downregulation of NEDD8 induced cell cycle arrest, DNA damage, and apoptosis in ESCC cells. Mechanistic studies demonstrated that NEDD8 knockdown led to the accumulation of cullin-RING E3 ubiquitin ligases (CRLs) substrates through inactivation of CRLs, thus suppressing the malignant phenotype by inducing cell cycle arrest and apoptosis in ESCC. Rescue experiments demonstrated that the induction of apoptosis after NEDD8 silencing was attenuated by DR5 knockdown.

**Conclusions:**

Our study elucidated the anti-ESCC effects and underlying mechanisms of NEDD8 knockdown, and validated NEDD8 as a potential target for ESCC therapy.

## Introduction

Esophageal carcinoma is a highly malignant gastrointestinal cancer accounting for almost half a million deaths per year worldwide^1^. Esophageal squamous cell carcinoma (ESCC) is the most common esophageal carcinoma histological subtype, and its incidence and mortality rates are rising rapidly^2^. The first-line treatment for ESCC is currently surgery in combination with neoadjuvant chemotherapy and radiotherapy; however, patients have poor prognosis, with a 5-year survival of 10%–15%^2,3^. Therefore, an effective therapeutic target for ESCC is urgently needed.

Neddylation is a protein posttranslational modification that covalently conjugates the ubiquitin-like molecule NEDD8 (neural precursor cell-expressed developmentally down-regulated) to substrate proteins through a series of enzymatic actions^4^. The cullin-RING E3 ubiquitin ligases (CRLs), the best characterized substrates of neddylation, affect many dynamic cellular processes, including the cell cycle, DNA damage, apoptosis, and tumorigenesis^5–8^. Moreover, members of the neddylation pathway, including NEDD8 and neddylation enzymes, are overexpressed in several types of cancers, and inhibition of the overexpressed neddylation pathway members may serve as a novel anticancer strategy^9–12^.

MLN4924 (Pevonedistat/TAK924) is a pharmaceutical inhibitor of NEDD8-activating enzyme E1 (NAE)^13^. Recently, a variety of phase II/III clinical trials of MLN4924 performed on human hematological malignancies and solid tumors have shown the efficacy of this treatment^14^. However, mutations in *UBA3* (a subunit of NAE E1), the target of MLN4924, have been found to lead to drug resistance^15,16^. Additionally, Zhou *et al.*^17,18^ have reported that MLN4924 stimulates tumor sphere formation, thus suggesting potential tumor promotion by MLN4924. The shortcomings of MLN4924 in cancer treatment underscore the need to identify other potential therapeutic targets against the neddylation pathway for anticancer therapy.

MLN4924 suppresses ESCC cell growth by targeting NAE E1^19,20^. However, whether other alternative targets against the neddylation pathway might be used in ESCC therapy warrants further exploration. In the present study, we demonstrate that overexpression of NEDD8 negatively correlates with overall survival in patients with ESCC. Furthermore, genetic downregulation of NEDD8 profoundly suppresses ESCC tumor growth by triggering cell cycle arrest, DNA damage, and apoptosis. Our study validates NEDD8 as an alternative therapeutic target against the neddylation pathway in ESCC therapy.

## Materials and methods

### Bioinformatics analysis of The Cancer Genome Atlas (TCGA) datasets

The mRNA expression level of *NEDD8* and corresponding clinicopathological data for esophageal carcinoma patients in the TCGA esophageal carcinoma dataset were collected from the UCSC xena website (http://xena.ucsc.edu/welcome-to-ucsc-xena/) and the web-portal UALCAN^21^. RNA-seq data for *NAE* and *UBC12*, as well as *NEDD8* gene mutations and amplifications, were downloaded from the TCGA esophageal carcinoma cohort. Survival was analyzed with the Kaplan-Meier method and compared with the log-rank test in Statistical Program for Social Sciences software (SPSS) Version 16.0. The association between copy number variation (CNV) frequencies and *NEDD8* gene expression was calculated with standard ANOVA and Tukey’s honest significant difference (HSD) tests.

### Immunohistochemistry and evaluation of human ESCC tissue arrays

Human ESCC tissue arrays, which purchased from Shanghai Outdo Biotech Co. Ltd. (Shanghai, China), were immunohistochemically stained with antibody to NEDD8 (Cell Signaling, Boston, MA). The tissue array sections were dehydrated and subjected to peroxidase blocking, then incubated with anti-NEDD8 at room temperature. The tissue array sections were then stained with a GTVisionTM III Detection System/Mo&Rb (Gene tech Company Limited) and counterstained with hematoxylin. The histologic evaluation was based on calculation of the percentage of positive tumor cells and the staining intensity, as described previously^22^. The detailed clinicopathologic characteristics of patients with ESCC are presented in **Supplementary Table S1**. This study was approved by the ethics committee of Huadong Hospital Affiliated to Fudan University (Approval No. 2016K007).

### Cell culture and generation of NEDD8-knockdown cells

The human esophageal epithelial cell line HET-1A and the human ESCC cell lines Kyse30, Kyse150, Kyse510, Kyse450 and EC1 were cultured in Dulbecco’s modified Eagle’s medium (Hyclone, Logan, UT, USA) containing 10% fetal bovine serum (Biochrom AG, Berlin, Germany) and 1% penicillin-streptomycin solution (Gibco, USA) at 37 °C under 5% CO_2_. Negative control and NEDD8-knockdown cells were established by infecting target cells with lentivirus particles packaged by HEK293T cells, which were co-transfected with the vector lenti-guide-puro (4.0 μg), and the packaging plasmids AGP091 (3.0 μg) and AGP090 (1.2 μg), with PEI (Polyfectine) reagent. Two different small guide RNA (sgRNA) oligonucleotides against NEDD8 were inserted into the lenti-guide-puro vector (4.0 μg). The human sgRNA sequences for *NEDD8* were as follows: 5′-ACCTGACTCACCTTGTCTGT-3′ and 5′-GAAGATGCTAATTAAAGTGA-3′. Targeted cells were seeded 1 day before infection with lentiviral supernatant along with 10 μg/mL polybrene (Sigma-Aldrich, St. louis, MO). Infected cells were selected with 2 μg/mL puromycin (Invitrogen, Carlsbad, CA) for 3 days.

### RNA extraction and real-time PCR

Cells were harvested, and total RNA was extracted with an Ultrapure RNA Kit from CWbiotech. Purified RNA (1.0 mg) was reverse transcribed with PrimeScript RT Master (Takara) according to the manufacturer’s instructions. Real-time polymerase chain reaction (PCR) was performed with SYBR Green Real-time PCR Master Mix (Applied Biosystems) on an ABI 7900 thermocycler (Thermo Fisher Scientific) according to the manufacturer’s instructions. For each sample, the mRNA abundance was normalized to that of *β-actin*. Sequences of primers were as follows: *β-actin*: forward, 5′-CCGTTGCCCTGAGGCTCTTT-3′, reverse, 5′-CCTTCTGCATCCTGTCAGCAA-3′; *NEDD8*: forward, 5′-AGACGCTGACCGGAAAGGA-3′, reverse, 5′-TCATCATTCATCTGCTTGCCAC-3′.

### Western blot and cycloheximide (CHX)-chase analysis

For Western blot, cell lysates (30 mg) were loaded on SDS-PAGE gels and transferred onto nitrocellulose membranes (Millipore), which were then incubated with the indicated primary antibodies overnight. Corresponding secondary antibodies were incubated with the membranes for 1 h, and the membranes were then photographed with a Tanon 5200 visualizer (Shanghai, China). Primary antibodies to the following proteins were used: UBC12, UBA3, cullin1, cullin2, cullin5, p21, NOXA (Abcam), NAE, cullin3, NEDD8, cullin4A, p27, Wee1, p-H3, ORC1, CDT1, p-H2AX, t-H2AX, ATF4, CHOP, DR5, cleaved-PARP, cleaved-caspase 8, and cleaved-caspase 3 (Cell Signaling, Boston, MA); cullin4B (Protein Tech); and β-actin (Protein Tech). For CHX-chase assays, cells were treated with 50 μg/mL CHX (Sigma) for the indicated times, and the band density in Western blot was quantified in Image J software.

### ATP-Lite cell viability assays

Cells were seeded in 96-well plates at a density of 1.5 × 10^3^ cells per well in triplicate and cultured for 72 h. Cell viability was determined with ATP-Lite luminescence assays (PerkinElmer, Norwalk, CT, USA) according to the manufacturer’s protocol.

### Colony formation assays

Cells were seeded into 6-well plates (300 cells/well) in triplicate, then incubated at 37 °C for 14 days. The 6-well plates were washed with cold phosphate-buffered saline 3 times and fixed with 4% paraformaldehyde at room temperature for 15 min. Colonies on the plates were stained with 0.1% crystal violet at room temperature for 30 min and then photographed under an inverted microscope (Olympus, Tokyo, Japan). Colonies with more than 50 cells were counted.

### Cell migration and invasion assays

Cells were prewashed twice and seeded at a density of 5 × 10^4^ cells per well in a 24-well Transwell polycarbonate filter plate (8 μm pore size; Corning, Lowell, MA). The upper Transwell chambers contained 200 μL serum-free medium, and the lower chambers contained 600 μL medium with 10% fetal bovine serum. In the invasion assays, a Matrigel polycarbonate membrane (Corning) was placed in the upper Transwell chamber. After 24 h incubation at 37 °C, the upper Transwell chambers were fixed in 4% paraformaldehyde for 20 min and stained with 0.1% crystal violet for 30 min. Cells on the outside of the top chambers were photographed and counted under an inverted microscope (Olympus, Tokyo, Japan).

### LC-MS/MS analysis and MS quantification

LC-MS/MS analysis and label-free based MS quantification of proteins in Kyse450 cells were performed as previously described^23^. A Firmiana proteomics workstation was used to process the raw MS data. Kyse450 lysates were digested into peptides with trypsin, then subjected to MS analysis on a Fusion Lumos instrument (Thermo Fisher Scientific). Raw MS data were used to interrogate the NCBI human Refseq protein database (released on 04-07-2013, 32,015 entries) with the Mascot search engine (version 2.3, Matrix Science Inc.) with a false discovery rate < 1% at the peptide and protein level. The intensity-based label-free quantification (iBAQ) algorithm was used for protein quantification. The iBAQ value was transferred into a fraction of total protein iBAQ amount per experiment (FOT). Then the FOT values were multiplied by 10^6^ and log_10_ transformed to obtain the FOT values for low abundance proteins.

### Flow cytometric analysis of the cell cycle and apoptosis

Cells were harvested with 0.25% trypsin without EDTA, then washed twice with cold phosphate-buffered saline. Cells used for cell cycle analysis were fixed in pre-cooled 70% ethanol at −20 °C overnight. Afterward, cells were stained with propidium iodide (PI, 36 μg/mL; Sigma, St. Louis, MO, USA) containing RNase A (10 μg/mL; Sigma, St. Louis, MO, USA) in the dark at 37 °C for 15 min, and detected with flow cytometry (BD FACSVerse™). Cells used in apoptosis assays were stained with Annexin V-fluorescein isothiocyanate (FITC) and PI with an Annexin V-FITC Apoptosis Detection Kit (Beckman Coulter) according to the manufacturer’s protocol, then subjected to flow cytometric analysis. Data were analyzed in FlowJo 7.6 software.

### Gene silencing with siRNA

Kyse450 cells with NEDD8 knockdown were transfected with the following siRNA oligonucleotides (synthesized by GenePharma, Shanghai, China) and Lipofectamine 2000 (Invitrogen, Carlsbad, CA, USA) reagent: *DR5*-1: 5′-AAGACC CUUGUGCUCGUUGUC-3′; *DR5*-2: 5′-CAGCCGUAGUCU UGAUUGUTT-3′; *NOXA*-1: 5′-GGUGCACGUUUCAUCAA UUUGTT-3′; *NOXA*-2: 5′-CCGGCAGAAACUUCUGAAUTT-3′; control: 5′-UUCUCCGAACGUGUCACGUTT-3′.

### Subcutaneous-transplantation tumor model

NC (negative control) or NEDD8-knockdown cells (5 × 10^6^ cells per mouse) were subcutaneously injected into 5-week-old BAL b/c female nude mice purchased from the Shanghai Lingchang Biotechnology Co., Ltd (Shanghai, China). Tumor size was measured with calipers at the indicated time points and calculated as (length × width^2^)/2. Mice were sacrificed at the end of the study, and tumor tissues were harvested, photographed, and weighed. Protein expression levels of the tumor tissues were evaluated with immunoblotting analysis with the indicated specific antibodies. Animal experiments were performed in accordance with the National Guidelines for Experimental Animal Welfare, with approval from the Institutional Animal Care and Use Committee of Fudan University (approval No. 202011008Z).

### Statistical analysis

The numerical results are presented as means ± standard deviations. Statistical significance for the comparison of parameters between 2 groups was evaluated with Student’s *t* test in GraphPad Prism5 software (GraphPad Software, Inc., San Diego, CA, USA). *P* < 0.05 was considered statistically significant, and n.s. denotes not significant. For all tests, 3 levels of significance (*P < 0.05, **P < 0.01, and ***P < 0.001) were used.

## Results

### NEDD8 overexpression is predictive of poor overall survival in patients with ESCC

**Figure 1:**
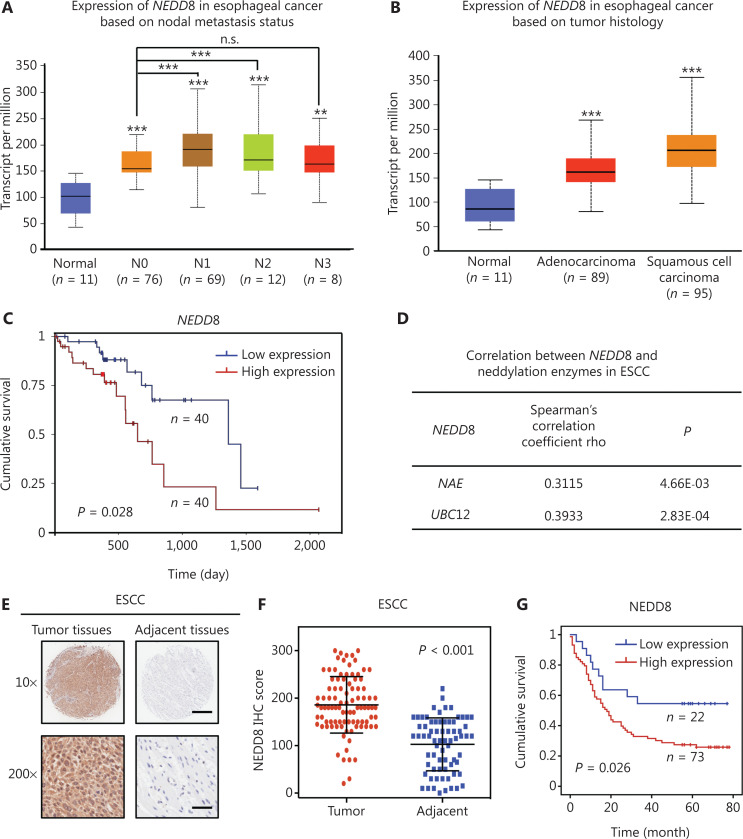
Overexpression of NEDD8 is predictive of poor overall survival in patients with ESCC. (A) The mRNA expression level of *NEDD8* was higher in esophageal carcinoma than in normal esophageal tissues. N0, N1, N2, and N3 denote nodal metastasis status. (B) The mRNA level of *NEDD8* was higher in ESCC and EAC than in normal esophageal tissues. (C) Kaplan-Meier curve analysis was performed to determine overall survival in patients with ESCC according to the *NEDD8* mRNA expression data in the TCGA RNA-Seq database. (D) The correlation between *NEDD8* and neddylation enzymes (*NAE* and *UBC12*) was analyzed in ESCC with the Spearman test. (E, F) A human ESCC tissue array was immunohistochemically stained with antibody specific to NEDD8 (E), and the difference in expression of NEDD8 in the ESCC tissues was calculated according to the histologic evaluation of tumors and adjacent normal tissues (F). Scale bar for 10× images, 500 μm; Scale bar for 200× images, 25 μm. (G) Kaplan-Meier curves based on NEDD8 protein expression in patients with ESCC. **P < 0.01, ***P < 0.001, n.s. = not significant.

To evaluate the clinical relevance of NEDD8 expression in esophageal carcinoma, we used the TCGA database to determine the levels of *NEDD8* transcripts in esophageal carcinoma. As shown in **Figure 1A**, the mRNA expression of *NEDD8* was significantly elevated in esophageal carcinoma and correlated with nodal metastasis (normal *vs.* N0, *P* = 2.5627E-04; normal *vs.* N1, *P* = 8.0956E-07; normal *vs.* N2, *P* = 5.5654E-08; normal *vs.* N3, *P* = 1.5798E-03; N0 *vs.* N1, *P* = 9.8279E-04; N0 *vs.* N2, *P* = 9.1616E-04; N0 *vs.* N3, *P* = 5.7208E-01). The NEDD8 mRNA expression in 2 histologic subtypes of esophageal carcinoma was significantly higher than that in normal tissues (normal *vs.* EAC: *P* = 1.5926E-05; normal *vs.* ESCC: *P* = 3.3506E-09) (**Figure 1B**). We further determined the association between the CNV frequencies and *NEDD8* gene expression by standard ANOVA and Tukey’s HSD tests. CNV gain was significantly associated with *NEDD8* gene expression in ESCC (*P* = 8.647E-04; **Supplementary Figures S1A and S1B**). These results indicated that the upregulation of *NEDD8* mRNA expression in ESCC might be partially explained by the CNV gain. Furthermore, Kaplan-Meier analysis showed that patients with ESCC with higher *NEDD8* expression had lower overall survival rates (*P* = 0.028, log-rank test; **Figure 1C**). Spearman tests indicated a statistically positive correlation between NEDD8 and the neddylation enzymes *NAE* and *UBC12* (*P* < 0.01; **Figure 1D**), thus suggesting that *NEDD8* expression and the neddylation pathway were positively correlated in ESCC.

In addition, we determined the NEDD8 protein expression in human ESCC tissue arrays through IHC. The NEDD8 protein expression was higher in ESCC tissues than in adjacent normal tissues (**Figure 1E**). Advanced histologic evaluation showed that the NEDD8 expression was significantly elevated in patient ESCC tissues (*P* < 0.001; **Figure 1F**). Furthermore, Kaplan-Meier analysis showed that patients with ESCC with high NEDD8 expression had poorer overall survival than those with low expression (*P* = 0.026, log-rank test; **Figure 1G and Supplementary Table S1**). Moreover, we detected the mRNA and protein levels of NEDD8 in the human esophageal epithelial cell line HET-1A and the 5 ESCC cell lines Kyse30, Kyse150, Kyse450, Kyse510 and EC1. The mRNA and protein levels of NEDD8 were higher in ESCC cell lines than in HET-1A (**Supplementary Figure S2A and S2B**). The above findings demonstrated that NEDD8 is overexpressed in ESCC and is associated with poorer overall survival, thus highlighting NEDD8 as a potential anti-ESCC target.

### NEDD8 knockdown suppresses the malignant phenotype of ESCC cells

**Figure 2:**
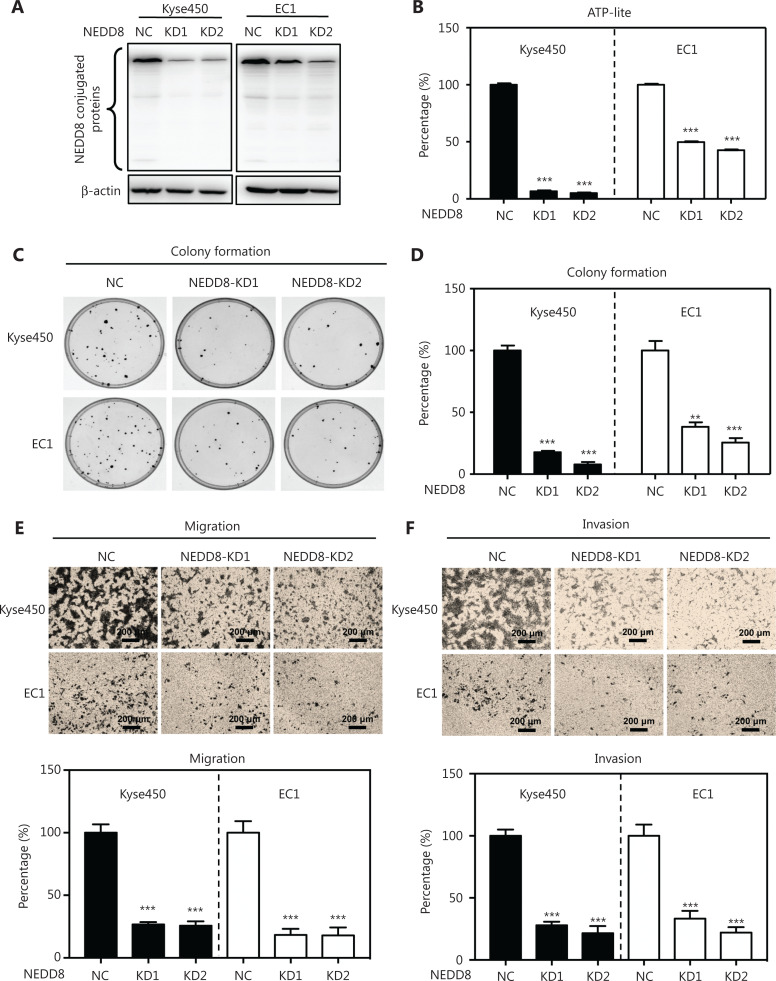
NEDD8 knockdown suppresses the malignant phenotype of ESCC cells. (A) Global protein neddylation levels were suppressed in NEDD8-knockdown Kyse450 and EC1 cells. Immunoblotting was used to analyze the global protein neddylation levels after NEDD8 knockdown, with β-actin as a loading control. (B) Cell viability analysis with ATP-Lite assays after NEDD8 knockdown. (C, D) Colony formation in NEDD8-knockdown Kyse450 and EC1 cells, determined by crystal violet staining and counting. Representative images are shown. (E, F) NEDD8-knockdown Kyse450 and EC1 cells were used to determine the migration and invasion abilities, as described in the Materials and methods. Representative images are shown; scale bar = 200 μm. Average values with standard deviations of triplicate experiments are shown. NC, negative control; KD, NEDD8 knockdown; **P < 0.01, ***P < 0.001.

To investigate the inhibitory effects of NEDD8 knockdown on the malignant phenotype of ESCC, we silenced NEDD8 with the CRISPR/Cas9 system in the ESCC cell lines Kyse450 and EC1 (**Figure 2A**). NEDD8 knockdown significantly inhibited the proliferation of these 2 ESCC cell lines (**Figure 2B and Supplementary Figure S3**). The colony formation abilities of these 2 cell lines were significantly repressed after NEDD8 knockdown (**Figure 2C and 2D**). Moreover, the Transwell migration and invasion abilities were significantly impaired in NEDD8-knockdown Kyse450 and EC1 cells (**Figure 2E and 2F**). These results demonstrated that downregulation of NEDD8 suppresses ESCC cell proliferation and survival, thus indicating that NEDD8 is necessary to maintain the malignant phenotype of ESCC cells.

### NEDD8 knockdown triggers multiple tumor-suppressive processes in ESCC cells

**Figure 3:**
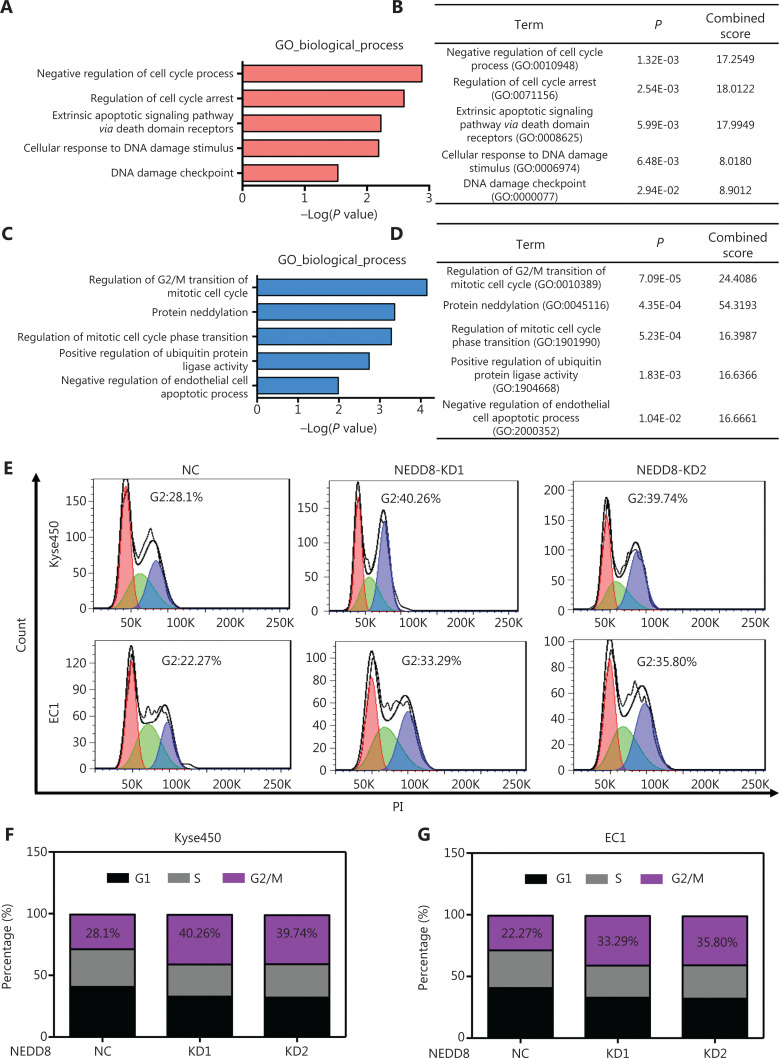
NEDD8 knockdown results in G2 phase cell cycle arrest. (A, B) Gene ontology (GO) analysis based on proteomics analysis was used to determine upregulated processes. (C, D) Downregulated biological processes were shown. (E–G) PI staining and FACS analysis were used to analyze the cell cycle profiles after NEDD8 depletion in Kyse450 and EC1 cells. NEDD8 knockdown induced ESCC cells arrest in G2 phase (presented in blue in E). The percentage of each cell cycle phase is shown in F and G.

To explore the mechanisms underlying the NEDD8 knockdown-induced cell malignant phenotype arrest, we performed quantitative proteomic analysis in NEDD8-knockdown Kyse450 cells. Gene ontology (GO) analysis indicated that several biological processes associated with cell survival—including negative regulation of cell cycle process, regulation of cell cycle arrest, extrinsic apoptotic signaling pathway *via* death domain receptors, cellular response to DNA damage stimulus, and DNA damage checkpoint—were significantly elevated in NEDD8-knockdown ESCC cells (**Figure 3A and 3B**). The regulation of G2/M transition in the mitotic cell cycle, protein neddylation, regulation of mitotic cell cycle phase transition, positive regulation of protein ubiquitin ligase activity and negative regulation of endothelial cell apoptotic process were significantly inhibited (**Figure 3C and 3D**). These findings were further supported by PI staining and fluorescence activated cell sorting (FACS) analysis, which showed that NEDD8 knockdown significantly increased the cell populations in the G2/M phase in both Kyse450 and EC1 cell lines (**Figure 3E–3G**). These results suggested that NEDD8 knockdown triggers DNA damage, cell cycle arrest, and apoptosis, thereby suppressing ESCC cell growth.

### NEDD8 knockdown triggers G2 phase cell cycle arrest due to the accumulation of CRL substrates in ESCC cells

**Figure 4:**
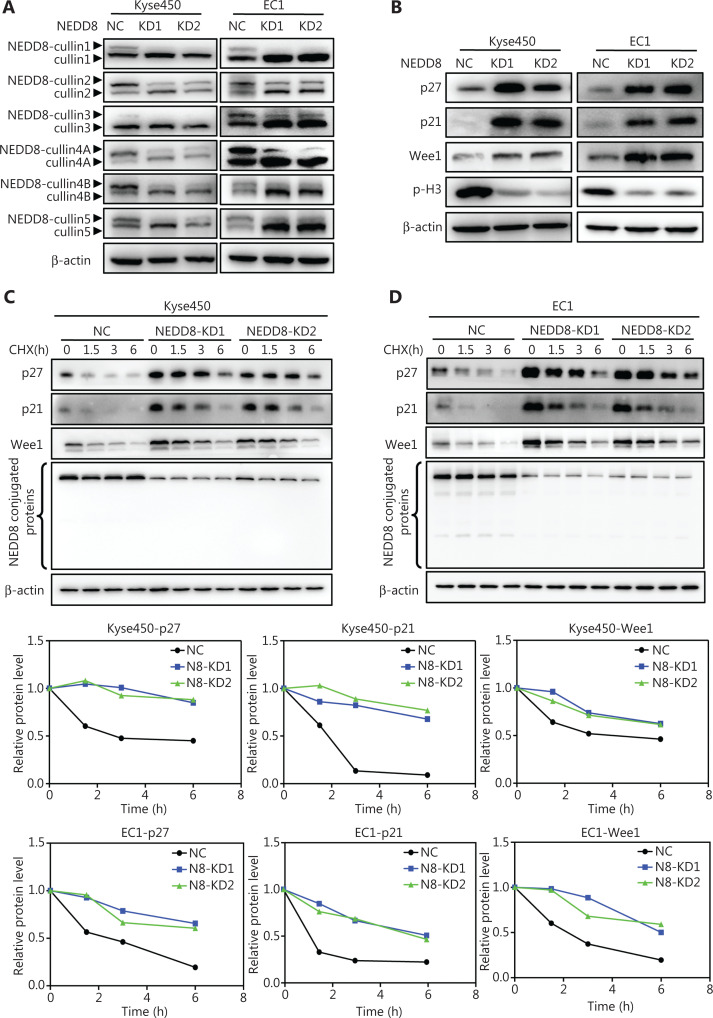
NEDD8 knockdown blocks the degradation of CRL substrates. (A) NEDD8 knockdown suppressed neddylation of cullin1, 2, 3, 4A, 4B, and 5 in Kyse450 and EC1 cells. (B) NEDD8 knockdown induced the accumulation of p27, p21, and Wee1, and a decrease in p-H3 in 2 ESCC cell lines. (C, D) The half-lives of p27, p21, and Wee1 were prolonged after NEDD8 knockdown in Kyse450 and EC1 cells. Cells were treated with CHX to block protein synthesis for the indicated times and then subjected to immunoblotting of p27, p21, and Wee1, with β-actin as a loading control. The protein levels of p27, p21, and Wee1 were quantified in comparison with β-actin, through intensity calculations in Image J software. NC, Negative control; KD, NEDD8 knockdown.

Because CRL E3 ligases are the best characterized substrates of neddylation, we determined the neddylation levels of cullins (the essential components of CRLs) after NEDD8 knockdown^7^. Immunoblotting analysis showed that downregulation of NEDD8 significantly decreased the neddylation levels of cullin1, 2, 3, 4A, 4B, and 5 (**Figure 4A**). However, NEDD8 knockdown had no effect on the expression levels of NAE1, UBA3, and UBC12 in both Kyse450 and EC1 cells (**Supplementary Figure S4**). Furthermore, the cell cycle inhibitors p21, p27, and Wee1, substrates of CRL E3 ligases, significantly accumulated after NEDD8 knockdown, whereas phosphorylated histone H3 (p-H3, ser10) was sharply downregulated (**Figure 4B**). These results were consistent with our above findings indicating that NEDD8-knockdown ESCC cells were arrested in G2 phase and did not enter M phase (**Figure 3E–3G**). To explore whether NEDD8 knockdown prolonged the half-lives of these cell cycle inhibitors as a result of CRLs inactivation, we used CHX to analyze the protein half-lives of p21, p27, and Wee1 after NEDD8 knockdown. As shown in **Figure 4C and 4D**, the half-lives of p21, p27, and Wee1 increased after NEDD8 knockdown. These results indicated that NEDD8 knockdown induces G2 phase cell cycle arrest, owing to the accumulation of the CRLs substrates p21, p27, and Wee1.

### NEDD8 knockdown induces DNA damage and activates DR5-dependent apoptosis in ESCC cells

**Figure 5:**
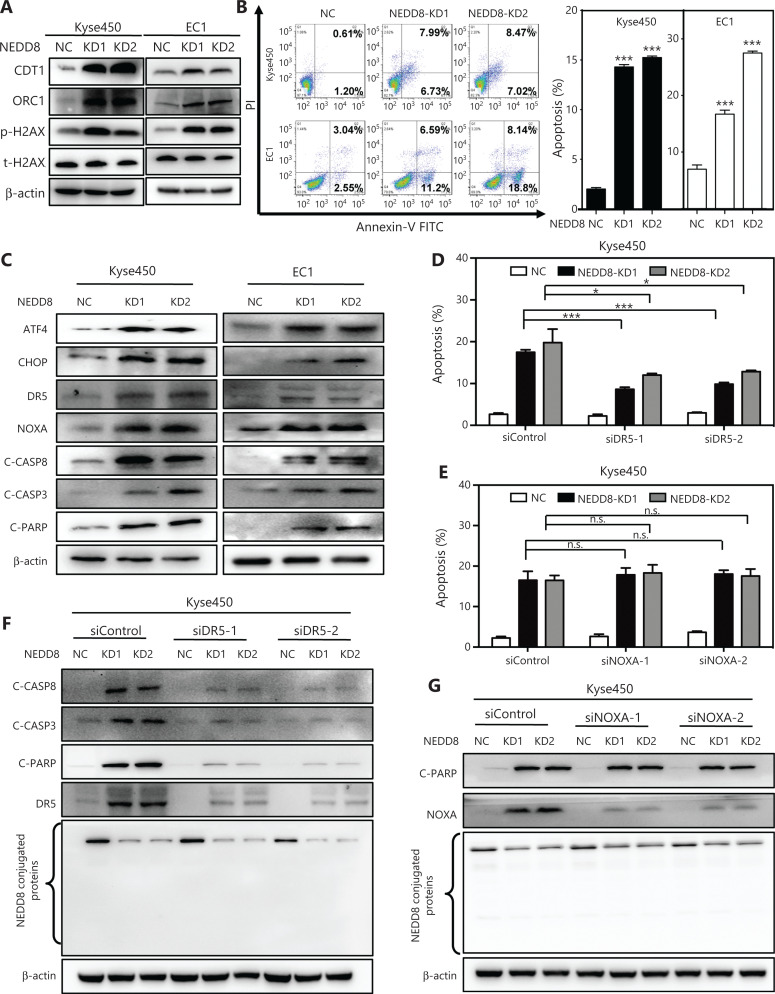
NEDD8 knockdown triggers DR5-dependent apoptosis in ESCC cells. (A) The expression levels of the DNA damage response proteins CDT1, ORC1, and phosphorylated/total H2AX in NEDD8-knockdown Kyse450 and EC1 cells were determined by immunoblotting, with β-actin as a loading control. (B) NEDD8 knockdown triggered apoptosis of ESCC cells, as determined by Annexin V–FITC/PI double-staining analysis. (C) NEDD8 knockdown induced accumulation of ATF4, CHOP, DR5, NOXA, cleaved-caspase 8, cleaved-caspase 3, and cleaved-PARP, as assessed by immunoblotting. (D) Downregulation of DR5 rescued apoptotic induction in NEDD8-knockdown Kyse450 cells. NEDD8-knockdown Kyse450 cells transfected with siControl or siDR5 and then subjected to Annexin V–FITC/PI double-staining analysis. (E) NOXA knockdown had no rescue effect on apoptotic induction in NEDD8-knockdown Kyse450 cells. siControl or siNOXA were transfected into Kyse450 cells, which were subjected to Annexin V-FITC/PI double-staining analysis. The cartogram showed no significance between 2 matching groups. (F) Kyse450 cells with siControl or siDR5 were subjected to immunoblotting for cleaved-caspase 8, cleaved-caspase 3, cleaved-PARP, and DR5, with β-actin as a loading control. (G) Kyse450 cells with siControl or siNOXA were subjected to immunoblotting of cleaved-PARP and NOXA, with β-actin as a loading control. Average values with standard deviations of triplicate experiments are shown. NC, negative control, KD, NEDD8 knockdown; *P < 0.05, ***P < 0.001, n.s. = not significant.

Our aforementioned GO analysis suggested that DNA damage effects and the apoptosis response were activated after NEDD8 knockdown (**Figure 3A–3D**). Therefore, we examined the DNA damage effects and apoptosis response in NEDD8-knockdown ESCC cells. As shown in **Figure 5A**, the DNA replication factor Cdt1 (CDT1), origin recognition complex subunit 1 (OCR1), and DNA damage marker phosphorylated H2AX accumulated after NEDD8 knockdown in both Kyse450 and EC1 cells. These results suggested that downregulation of NEDD8 induced DNA re-replication stress and subsequent DNA damage. NEDD8 knockdown also resulted in a shrunken morphology of apoptotic cells in both Kyse450 and EC1 cell lines (data not shown). Consistently, the Annexin V-positive cell populations significantly increased after NEDD8 knockdown in both cell lines, thus indicating that NEDD8 knockdown increased the apoptotic response of ESCC cells (**Figure 5B**). Further mechanistic studies showed that NEDD8 knockdown induced accumulation of the CRLs substrate activating transcription factor 4 (ATF4) and the important apoptosis mediator phorbol-12-myristate-13-acetate-induced protein 1 (NOXA), as well as the classical apoptotic hallmarks cleaved-caspase 3 and cleaved-PARP (**Figure 5C**). After ATF4 accumulation, the transcription factor C/EBP-homologous protein (CHOP), a classical downstream target of ATF4, was activated and further induced the expression of DR5, which in turn activated cleaved-caspase 8 and triggered extrinsic apoptosis (**Figure 5C**). To further explore the mechanisms underlying the apoptosis induced by NEDD8 knockdown, we knocked down NOXA and DR5 with siRNA silencing. Only DR5 knockdown resulted in a partial rescue of apoptosis induced by NEDD8 knockdown (**Figure 5D and 5F, Supplementary Figure S5**). In contrast, NOXA knockdown did not rescue the apoptotic induction in NEDD8-knockdown Kyse450 cells (**Figure 5E and 5G**). These results indicated that NEDD8 deficiency induces DNA damage and triggers DR5-dependent extrinsic apoptosis in ESCC cells.

### NEDD8 knockdown suppresses ESCC tumor growth *in vivo*

**Figure 6:**
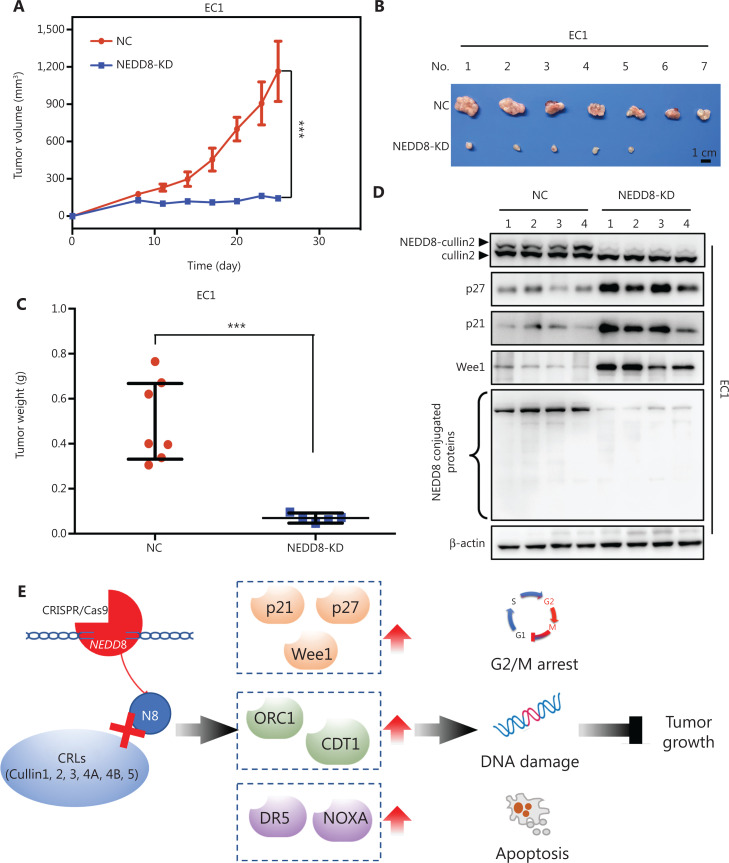
NEDD8 knockdown suppresses ESCC tumor growth *in vivo*. The subcutaneous-transplantation tumor model was established by using NEDD8-knockdown EC1 cells. (A) Tumor size was measured with calipers at the indicated time points and converted to a tumor growth curve. (B) Tumor tissues were harvested and photographed on the day of sacrifice. Scale bar = 1 cm. (C) The tumor weight was measured. (D) Proteins extracted from tumor tissues were analyzed by immunoblotting against cullin2, p27, p21, Wee1, and NEDD8, with β-actin as a loading control. (E) Working model in which NEDD8 knockdown suppresses the tumor growth of ESCC. NC, negative control, KD, NEDD8 knockdown; ***P < 0.001.

To investigate the therapeutic potential of NEDD8 silencing *in vivo*, we established a subcutaneous-transplantation tumor model using NEDD8-knockdown EC1 cells. Downregulation of NEDD8 significantly inhibited tumor growth, according to the tumor growth curve (*P* < 0.001; **Figure 6A**). Moreover, compared with the control group mice, which developed 100% (7/7) large tumors, only 71.4% (5/7) of the NEDD8-knockdown group mice developed small tumors (**Figure 6B**); these results were further supported by tumor weight assessment (*P* < 0.001; **Figure 6C**). We further addressed the potential mechanisms underlying the antitumor activity of NEDD8 knockdown *in vivo*. Downregulation of NEDD8 profoundly inhibited cullin neddylation, thus indicating the inactivation of CRLs. Consequently, the CRLs substrates p27, p21, and Wee1 clearly accumulated in the NEDD8-knockdown group (**Figure 6D**). Collectively, our results indicated that knockdown of NEDD8 leads to CRLs inactivation and the accumulation of tumor-suppressive CRL substrates, thus inhibiting tumor growth *in vivo*.

## Discussion

ESCC is one of the deadliest digestive system cancers worldwide^24^. However, effective therapeutic strategies for ESCC are lacking^25,26^. Therefore, developing novel and effective anti-ESCC therapeutic targets is urgently needed. The neddylation pathway is overactivated in multiple human cancers, and inhibition of the neddylation pathway by MLN4924 has provided an attractive anticancer therapeutic strategy^27^. However, drug resistance and potential tumor promotion of MLN4924 have led to a demand for new neddylation pathway anticancer targets^15,28^. In the present study, we demonstrated that NEDD8 was elevated in ESCC, and patients with high expression of NEDD8 had poorer overall survival. We determined the potential of NEDD8 as an alternative therapeutic target for ESCC therapy. Our results showed that downregulation of NEDD8 activated a series of biological tumor-suppressive processes. Furthermore, NEDD8 silencing displayed anticancer effects *in vitro* and *in vivo*, thus confirming NEDD8 as a potential effective therapeutic target for ESCC (**Figure 6E**).

A previous study has reported that NEDD8 is upregulated in multiple cancers, and NEDD8 overexpression is associated with poor prognosis^29^. In the present study, we showed that the mRNA and protein expression levels of NEDD8 were significantly elevated in ESCC, and overexpressed NEDD8 negatively correlated with overall survival in patients with ESCC, thus indicating that NEDD8 may be a useful biomarker for ESCC. In addition, NEDD8 knockdown not only obviously suppressed ESCC cell proliferation but also profoundly inhibited the migration and invasion abilities of ESCC cells. *In vivo* experiments further showed that downregulation of NEDD8 significantly repressed ESCC tumor growth. Therefore, our study suggests that NEDD8 may be a promising therapeutic target and provides a scientific basis for the development of specific inhibitors targeting NEDD8.

Mass spectrometry analysis identified 1219 proteins up-regulated > 2-fold and 1108 proteins down-regulated < 2-fold in NEDD8-knockdown ESCC cells. On the basis of our quantitative proteomic analysis results, we demonstrated that downregulation of NEDD8 affects the cell cycle. Mechanistically, we found that NEDD8 knockdown inactivates CRLs and induces the accumulation of cell cycle inhibitor proteins (p21, p27, and Wee1) in ESCC cells. Accordingly, cell cycle progression is blocked. Proteomic analysis also indicated that downregulation of NEDD8 induced extrinsic apoptosis in ESCC cells. Indeed, knockdown NEDD8 induced extrinsic apoptosis *via* the accumulation of DR5, owing to the upregulation of ATF4, a substrate of CRL1, and its downstream target transcription factor CHOP^30^. Further rescue experiments suggested that NEDD8 deficiency induced extrinsic apoptosis in a manner dependent on DR5 in ESCC cells. Therefore, our results demonstrated that genetic downregulation of NEDD8 has anti-ESCC effects and mechanisms similar to those of MLN4924, and confirmed NEDD8 as a therapeutic target in ESCC.

## Conclusions

Overall, our findings not only validate NEDD8 as a promising therapeutic target against ESCC but also provide a basis for NEDD8 inhibitor development. We believe that the development of novel anti-ESCC strategies targeting NEDD8 would shed new light on ESCC treatment.
