# Supplementary material for: Overexpressed NEDD8 as a potential therapeutic target in esophageal squamous cell carcinoma
Source: Cancer Biol Med. 2021 Mar 18;19(4):504–17. doi: 10.20892/j.issn.2095-3941.2020.0484 (PMC9088192; doi:10.20892/j.issn.2095-3941.2020.0484)
Supplement: Supplementary file 1 [file cbm-19-504-s001.pdf]

Supplementary materials

A

Copy number variation frequencies of *NEDD8* in ESCC

Gene	CNV gain	CNV loss
NEDD8	12/95 (12.60%)	8/95 (8.42%)

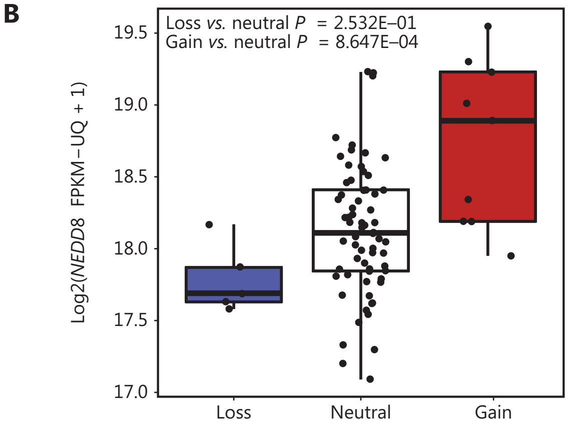

Figure S1 The copy number variation (CNV) frequencies of *NEDD8* in ESCC, calculated from the TCGA database. (A) The CNV gain for *NEDD8* in ESCC was 12.60% and the CNV loss for *NEDD8* was 8.42%. (B) The association between CNV and *NEDD8* gene expression was calculated with standard ANOVA and Tukey's HSD tests.

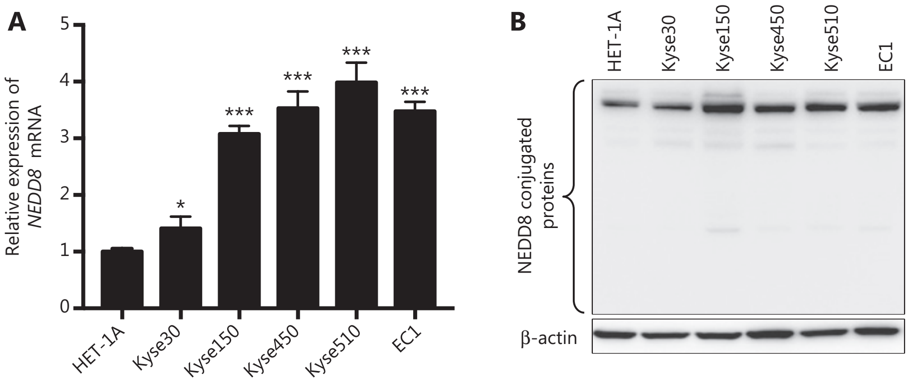

Figure S2 The mRNA and protein levels of NEDD8 are higher in ESCC cell lines than in non-cancerous cells. (A) mRNA and (B) protein expression levels of NEDD8 in HET-1A, Kyse30, Kyse150, Kyse450, Kyse510, and EC1 cell lines, determined with real-time PCR and immunoblotting, respectively. *$P < 0.05$, ***$P < 0.001$.

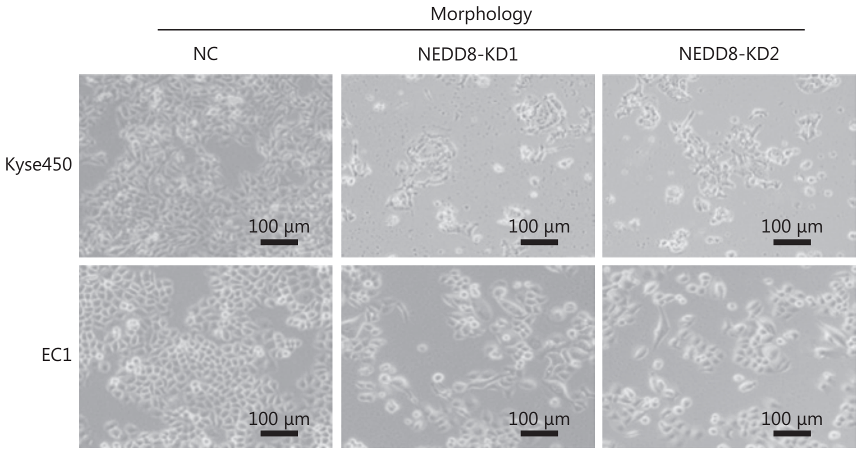

Figure S3 NEDD8 knockdown suppresses the growth of the Kyse450 cells and EC1 cells. Representative micrographs of NEDD8-knockdown Kyse450 and EC1 cells are shown; scale bar = 100 μm.

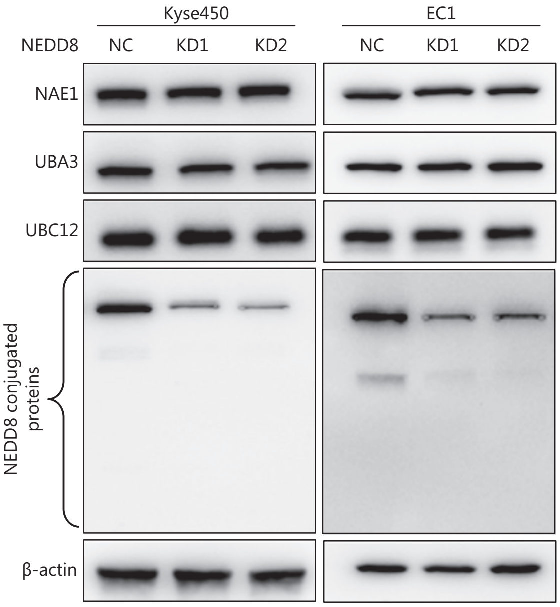

Figure S4 NEDD8 knockdown does not affect the protein levels of neddylation enzymes. The expression levels of NAE1, UBA3, and UBC12 in NEDD8-knockdown ESCC cells were determined by immunoblotting.

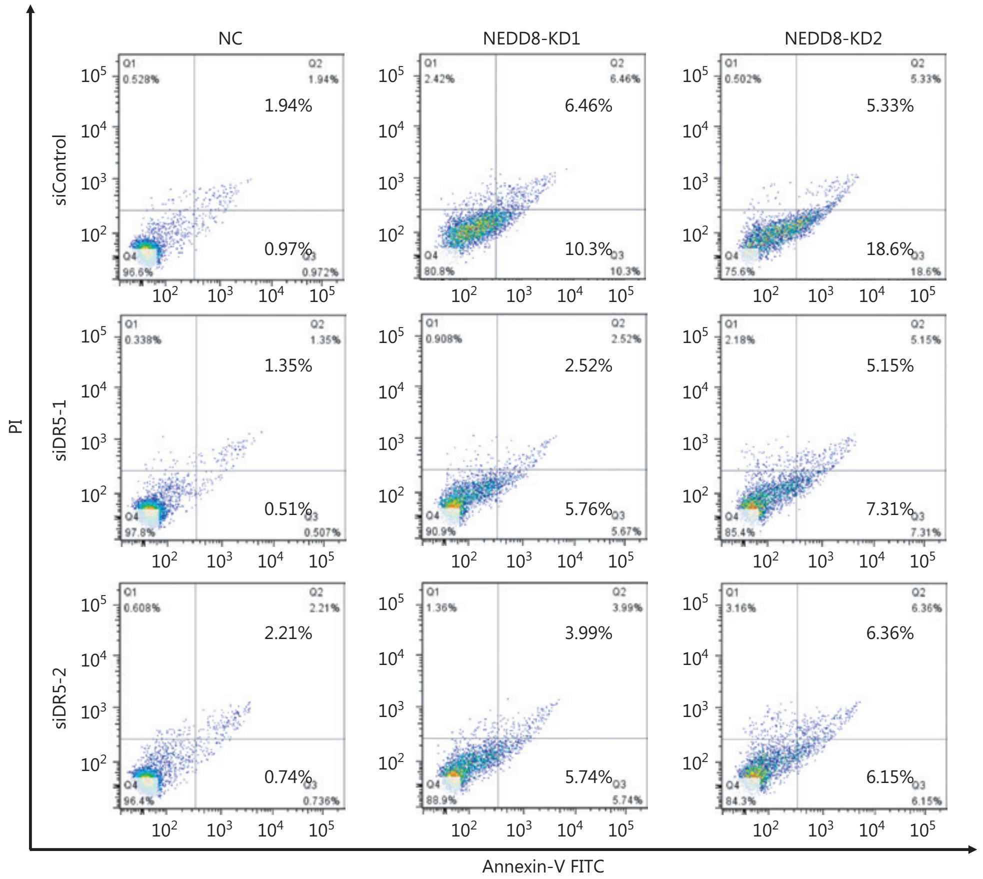

Figure S5 Downregulation of DR5 rescues the apoptosis induced by NEDD8 knockdown in Kyse450 cells. NEDD8-knockdown Kyse450 cells transfected with siControl or siDR5 were subjected to Annexin V–FITC/PI double-staining analysis.

Table S1 Clinicopathologic parameters according to the expression of NEDD8

Variable	Overall No.	NEDD8		*P*
		Low, *n*	High, *n*	
Age (n = 95)				0.956
< 60 years	22	5	17	
≥ 60 years	73	17	56	
Gender (n = 95)				0.256
Male	77	16	61	
Female	18	6	12	
Tumor (n = 88)				0.052
T1	3	2	1	
T2	19	1	18	
T3	64	17	47	
T4	2	0	2	
Node (n = 95)				0.820
N_0	47	9	38	
N_1	28	8	20	
N_2	16	4	12	
N_3	4	1	3	
TNM (n = 88)				0.704
I	6	2	4	
II	39	7	32	
III	37	10	27	
IV	6	1	5	
